# Cemented dual mobility cup for primary total hip arthroplasty: survival and quality of life. A multicenter study

**DOI:** 10.1051/sicotj/2025006

**Published:** 2025-03-13

**Authors:** Jairo Alonso Rincón, Camilo de la Pava, Rubén Velandia, Sofía Muñoz-Medina, Andre Ferreira

**Affiliations:** 1 Area of Orthopedic Surgery and Traumatology, Sanitas Organización Internacional, Grupo Keralty Bogotá 111321 Colombia; 2 Unidad de Investigación, Fundación Universitaria Sanitas Bogotá 111321 Colombia; 3 Universidad Nacional de Colombia Bogotá 111321 Colombia; 4 Area of Orthopedic Surgery and Traumatology, Clinique du Parc Lyon Lyon 69006 France

**Keywords:** Hip, Replacement, Arthroplasty, Hip prosthesis, Survival, Quality of life, Cemented dual mobility cup

## Abstract

*Introduction:* Dual mobility cups are characterized by having a prosthetic head inside a polyethylene core that later articulates with a metal cup implanted in the acetabulum. These cups can be cemented or uncemented. This study aimed to determine the survival of a cemented dual-mobility cup (CDMC) with a cobalt-chromium head (CoCr) and the quality of life (QOL) of operated patients. *Methodology:* Multicenter historical cohort study where survival and QOL were estimated. The cohort includes patients who underwent a primary total hip arthroplasty (THA) with a CDMC and CoCr head. The patients were operated on between 2011 and 2013. *Results:* 40 patients from 6 institutions with a median age of 81 (IQR 22.25) years. The results in the Kaplan-Meier estimation showed a survival of 94.2% (95% CI [86.6% – 100%]) at 5 years and a maximum follow-up of 9.5 years. Three failures occurred (two dislocations and one mechanical loosening), and Oxford Hip Scale (OHS) of 41.5 (IQR 10.50) points was recorded. *Conclusions:* In terms of survival and the score obtained in the OHS, the CDMC has comparable results with the scientific literature found on uncemented dual mobility cups. This demonstrates adequate results in patients with a maximum follow-up of 9.5 years.

## Introduction

Osteoarthritis (OA) had a growing incidence in recent years. Worldwide it went from 13.75 in 1990 to 20.48 cases per 100,000 persons in 2019. In Colombia, it went from 7.69 to 15.47 per 100,000 persons for the same period, while in high-income countries the incidence is higher [1]. OA is the most common cause of disability in older adults [2] and the initial treatment can be based on individualized self-care strategies that generate healthy habits such as exercise, weight loss, the use of appropriate footwear, and step counting [3], however, when these strategies do not work and the OA is in an advanced stage, a total hip arthroplasty (THA) is required.

Dual mobility cups were designed in France by Gilles Bousquet in 1974 [4]. There is extensive experience with these cups in Europe [5], however, the use of this type of device has been debated for years, and currently, there is still controversy in using these as an option in primary THA surgeries [6], nevertheless, the American Joint Replacement Registry and the Joint National Registry in the United Kingdom show an increase in the use of dual mobility cups for primary and revision surgeries [7, 8].

THA is currently trending upwards, according to reports from the American Joint Replacement Registry [7], the United Kingdom Joint National Registry annual report [8], and the National Association of Joint Replacement Registry Australian Orthopedics [9]. One of the main complications and indications for revision surgery in THA is instability and/or dislocation, as confirmed by records from countries such as Norway, Sweden, Australia, the United Kingdom, and the United States [7–11], a possible solution for this complication is the use of dual mobility cups, according to numerous studies [12–16]; this type of cup offers a safe, effective, and long-lasting solution to hip instability. Additionally, the dual mobility system allows for a range of motion that exceeds traditional hip prosthesis while maintaining the integrity of the device [5].

The study developed by Rincón Hoyos et al. [17] made a first approximation to the survival and indications of the Cemented Dual Mobility Cup (CDMC) in the Colombian context, likewise, they measured the quality of life (QoL) in patients with this device, this study was proposed as a continuity with the 10-year follow-up of the patients, the objective was to determine the survival of the CDMC with cobalt-chromium head (CoCr) and to estimate the QoL of the patients who underwent a primary THA.

## Methods

Analytical observational multicenter study of historical cohort type, with patients who underwent implantation of a QUATTRO™ reference of CDMC manufactured by the Groupe Lepine from France with posterolateral surgical approach, no other type of cup was included. The patients were operated on by five surgeons in six clinics.

Patients who were older than 18 years, with primary THA using CDMC, either with cemented or cementless stems, and a CoCr head implanted between 2011 and 2013, the indications were: osteoarthritis, fracture, avascular necrosis, or neoplasms were included. Patients with uncontrolled lumbar and psychiatric pathologies that did not allow the application of the Oxford Hip Scale (OHS) were excluded. Additionally, the following variables were measured: age, life status, gender, laterality of the operated hip, indication for primary surgery, surgery approach, revision required, and its indication.

Ethically, this project followed the Helsinki Declaration and maintained ethical principles and the confidentiality and anonymization of patient data. This study was approved by the institution’s ethics committee in July 2021.

### Measured results

Survival was estimated using the Kaplan-Meier (KM) method, where the initial time was the moment of CDMC implantation between 2011 and 2013. Failure was defined as the moment in which revision surgery was required, however, in cases of revision surgery exclusive to the femoral component, it was not taken as an event in the survival analysis. QoL was measured through the OHS, validated in Spanish and for Colombia by Martínez et al. [18]. In the cases where the patients had revision surgery, the search and reading of the clinical history and radiological images were carried out.

### Statistical analysis

A descriptive analysis was carried out to characterize the population, and the quantitative variables were described with measures of central tendency and dispersion, according to the Shapiro-Wilk test, given the low number of patients it was considered that the data did not come from a normal distribution, reporting the median and interquartile range (IQR), qualitative variables using absolute frequencies and percentages. Survival analysis was performed using the KM estimation method with a 95% confidence interval, the analyses were performed using R.

## Results

The historical cohort analyzed had a total of 40 patients, the characteristics can be seen in Table 1.

**Table 1:** Primary THA with CDMC.

Variable	Categories	Median	I.Q.R.
Age	Years	81	22.25
Variable	Categories	*n*	%
Live-status	Live	12	30.00%
	Die	28	70.00%
Gender	Female	31	77.50%
	Male	9	22.50%
Laterally	Right	17	42.50%
	Left	23	57.50%
Indication for primary surgery	Osteoarthritis	19	47.50%
	Fracture	19	47.50%
	Avascular necrosis	1	2.50%
	Bone neoplasms	1	2.50%
Revision surgery	Required	3	7.50%
	Not required	37	92.50%
Indication for revision surgery	Dislocation	2	66.67%
	Mechanical loosening	1	33.33%

**Figure 1:**
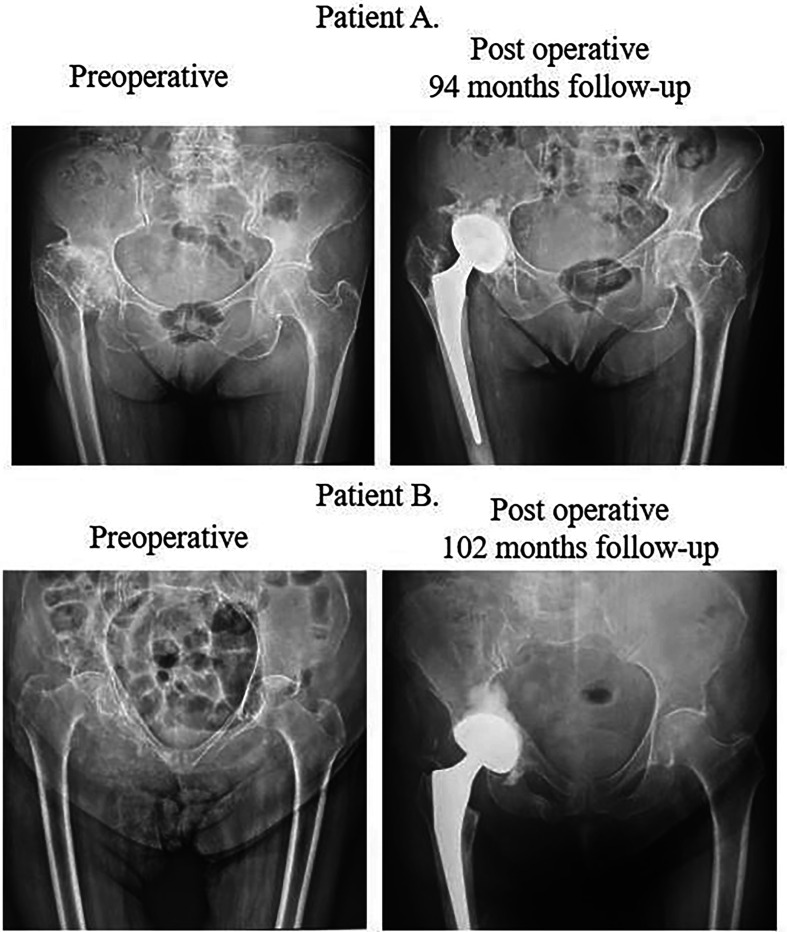
Radiographs of two patients.

About 77.5% of the procedures were performed with a posterolateral approach and 22.5% with an anterolateral approach. 95% of the indications for surgery were OA and fractures. Preoperative and postoperative radiographs of two cases are shown in Figure 1.

**Figure 2:**
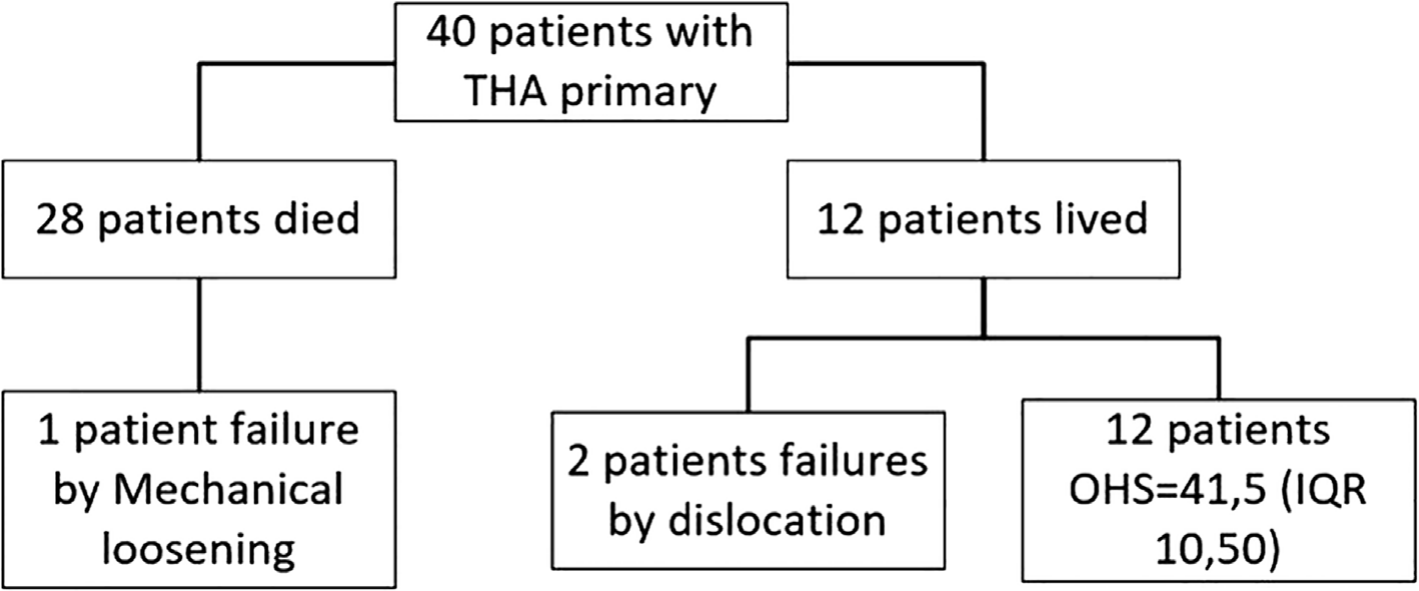
Patient flowchart.

Three patients required revision with an incidence of 7.5%, two due to dislocation, one of these cases suffered dislocation in the first month during muscle healing, and the other one occurred four years after surgery during an extreme range of motion of the hip, and 1 patient due to mechanical loosening. The follow-up of the included patients can be seen in Figure 2.

**Figure 3:**
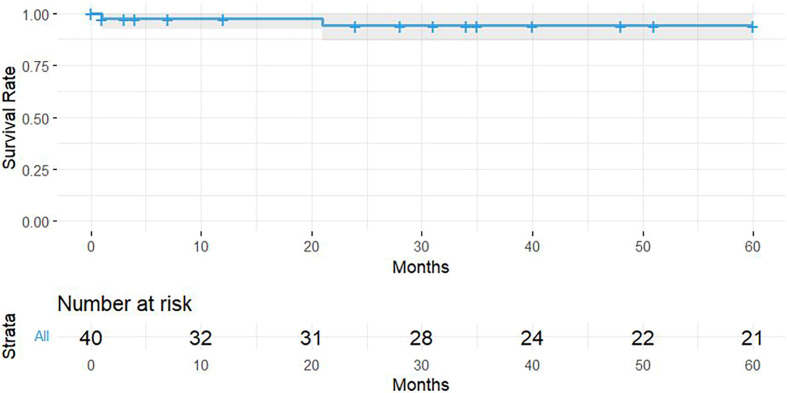
Survival and follow-up of CDMC with CoCr head.

In the initial exploration of the data, the Shapiro-Wilk normality tests were performed, however, normality was not assumed for the analysis of the OHS results due to the decrease in the sample size because 70% of the patients died. The median follow-up time was 5.33 (IQR 5.92) years and the results in the OHS were 41.5 (IQR 10.50) points applied to 12 living patients. The score obtained in the OHS is in the category of excellent according to the classification developed by Kalairajah et al. [19]. In the KM, survival was 94.2% (95% CI [86.6% – 100%]) at 5 years of follow-up presented in Figure 3. Due to the retrospective methodology used, there were no losses to follow-up. The maximum follow-up period was 9.5 years, with 1 out of 3 cases of revision reported within 5.3 years.

## Discussion

CDMC is indicated in patients with poor bone quality, for example, in patients with severe osteoporosis or autoimmune diseases, or in those patients where an adequate ring for fixation is not achieved (press fit) of a cementless dual mobility cup, according to the indications described in the study published by Rincón Hoyos et al. [17]. It’s important to recognize the impact in years lived with a disability of low bone mineral density is higher in France if compare with Colombia (20), this trend may explain the low indication of CDMC in the Colombian population and the low size of the sample takes on more importance. In Rincón Hoyos et al. [17] CDMC survival of 97.6% was reported at 2.6 years of follow-up, in this study where the same cohort of patients was analyzed, survival decreased to 94.2% (95% CI [86.6% – 100%]) at 5 years, however, there was a large percentage of patients who died, this may be related to life expectancy in Colombia, which is 77, 2 years [20] and the median age of the patients was 81 years, this being higher than life expectancy.

The study by Philippot et al. [21] reported a 15-year survival rate of 96.3 ± 3.7% for cementless dual mobility cups, however, the mean age of the patients was 54.8 years (range 23–87) and two types of cementless cups were used. In the article by Prudhon et al. [22], the survival of cementless dual mobility cups was 95% with a 95% CI in the range of 81.5–98.8 and the average age of the population was 78 years (range 39.8–93, 5). Survival in our study is between 5 and 6 percentage points lower than expected; however, the comparison is questionable since it was made with cementless cups and for nonequivalent patients.

The QOL reported in the study by Matsen et al. [23] reports an average OHS score of 43 (range 13 – 48) with an average follow-up time of 27.6 months (range 21 – 38), in the studies by Chouteau et al. [24] with 8.4 years (range 7–11) of follow-up report an OHS of 40.3 ± 6.7 and Fessy et al. [25] with 8.7 years (range 6.8–10.5) of follow-up report an OHS of 41.2 ± 7.6, these last three studies were performed with uncemented dual mobility cups, however, our results were similar to those reported with 41.5 points in the OHS.

Systematic review and meta-analysis developed by Albanese et al. [26] the cumulative incidence of dislocation for dual mobility cups was estimated at 1.2 (95% CI: 0.3% – 2.7%), however, in the included study with the largest sample size by Tabori-Jensen et al. [27] with a follow-up of 1.6 years, reported 4.7% dislocations and 0.8% revision surgeries. In our study, two cases of dislocation were reported corresponding to an incidence of 5%, this value is comparable to that reported by Tabori-Jensen et al. [27] but not with the values reported in the forest plot of dislocations in the study by Albanese et al. [26], nevertheless, in our study two out of three cases that failed could explain by their indication for surgery was hip fracture where the risk of dislocation increases up to five times [4], the posterior surgical approach has a significant difference in favor of the anterior approach in terms of length of stay and dislocations, however, the surgical approach was used was posterolateral, finally, the other failure was mechanical loosening.

The CDMC survival of 94.2% was adequate for the analyzed follow-up time and there are similar findings with the scientific literature found, the score obtained in the OHS of 41.5 is comparable with those reported, however, most comparisons were made with studies that include cementless dual mobility cups, it is necessary to carry out a greater number of studies in CDMC. Xiao et al. [28] confirmed the prevalence of osteoporosis analyzed by continent is higher in America at 17.8% compared to Europe at 14.4%, severe osteoporosis could be a justification for using a cemented implant.

This study shows the continuation of the follow-up of a cohort of patients in the context of the Colombian health system and is very useful for decision-making and obtaining better results in patients with advanced-stage OA, however, the study’s limitation was a high percentage of deaths and its retrospective design.

## Conclusion

Our study shows that CMDC could be an acceptable procedure when bone status doesn’t allow a press fit with a cementless fixation, this intervention may help patients suffering from osteoarthritis.

## Data Availability

Data will be available upon request. Please contact the corresponding author.
